# Treatise on Surgical Anatomy, Etc.

**Published:** 1860-09

**Authors:** 


ART. VI.—*Traité d'Anatomie Chirurgicale et de Chirurgie Expérimentale.* Par J. F. MALGAIGNE, M.D., Professeur, etc.

*Treatise on Surgical Anatomy and Experimental Surgery.* By J. F. MALGAIGNE. Two volumes, 8vo., pp. 784–880. Paris, 1859.

MONS. MALGAIGNE needs no special introduction to our readers, as his name has long been familiar to the profession on this side of the Atlantic. His great treatise on Fractures and Dislocations, the first volume of which was so ably translated and edited a few years ago by Dr. Packard of this city, his Operative Surgery, his celebrated edition of the works of Ambrose Paré, and his various contributions to the periodical press, especially his papers on the statistics of hernia, have been widely circulated in this country, and have given him an American reputation. A professor in the School of Medicine of Paris, he is one of the surgeons of the

Beaujon Hospital, a member of the Imperial Academy of Medicine, and an officer of the Legion of Honor; positions which he has fairly won by his indomitable industry, his eminent talents, and his faithful services in the cause of science and humanity. The present edition of the Surgical Anatomy is the second, the first having been published nearly twenty years ago, and soon after reprinted at Brussels, whence many copies found their way into the United States. It has been thoroughly revised and greatly enlarged, the principal additions having been made to the chapters on the eye, general anatomy, the arteries and veins, the nervous system, and the history of the blood. The sections on special anatomy, hernia, luxations, and fractures have also been greatly extended; in fact, nearly all of them have been completely remodeled, and enriched with the results of new dissections, experiments, and deductions. The work, in its present garb, is an immense storehouse of facts and erudition, and will prove an enduring monument to the author's talents, industry, and research. Yet, notwithstanding this, it is anything but a great work on surgical anatomy; it takes in entirely too much ground; it is spread over too great a surface; in short, it is diluted with all things relevant and irrelevant; and had M. Malgaigne written a work on anthropology, he could not have missed his aim more sadly or more effectually. When we declare that he has incorporated in the present edition an immense amount of matter on general anatomy, and upwards of one hundred and fifty pages on the physical, chemical, and vital properties of the blood, the reader will agree with us that he has strangely, and, as far as the legitimate topics of his treatise are concerned, most unfortunately wandered from his subject. Surgical anatomy requires no such aids or extrinsic embellishments. The facts with which it deals are of a more circumscribed nature, and hence all such material only serves to clog and hinder it. Whether we apply to it the term surgical, topographical, or regional, it is of no consequence in a practical sense; it treats of one thing, and of one only, namely, the mutual relations of parts, or the relative position which one structure bears to another, considered in its application to surgical operations and accidents. Whatever, therefore, is foreign to these points, is so far foreign to the subject, legitimately considered; and regarded in this light, the work of Malgaigne must be viewed as a prodigious failure. No man living, unless it be the author himself, will ever think of wading through such a wilderness of matter to obtain a just idea of surgical anatomy; and yet, as was stated previously, the work is a great work, destined to live as long as anatomical science shall have a place in human pursuits. The title is evidently a misnomer: it should be, not a treatise on surgical anatomy and experimental surgery, but a treatise on histology, surgical anatomy, and surgical pathology

We have no disposition, after what has been stated, to give an analysis of this production; to do so would far exceed the limits which we could afford to such a subject. Let those who feel inclined obtain the work, and study it in detail, or such portions of it as their peculiar wants may require. As a book of reference, it is invaluable; as a book to work by in the dissecting-room, detestable.

The history of surgical anatomy is not without interest. It is of modern date, extending no farther back than the commencement of the present century. John Palfin, who died in 1730, had, it is true, some faint conceptions of it, but they were obscured by so many clouds that they failed to make any impression whatever upon his contemporaries and immediate successors. Desault, one of the representative men of his age, was the first, toward the close of the eighteenth century, to give regular and systematic courses of lectures on surgical anatomy, but as he never wrote upon the subject, the influence of his teaching must have been limited, in great degree, to his own countrymen and to the few foreigners who resorted for instruction to the French capital. The "old cur," as he was familiarly called by his pupils, never published anything except a few detached papers; and had it not been for his gifted friend and great disciple, Bichat, who was destined to play so conspicuous a part in the science of general anatomy, of which he was at once the founder and the greatest promoter, even the clinical labors of Desault would probably have been lost to the world.

The first professed work on surgical anatomy of which we have any knowledge was published in 1811, at Weimar, by J. C. Rosenmüller, the author of a small but popular work on descriptive anatomy, and the discoverer of the little muscle connected with the lachrymal sac, afterwards described by the late Professor Horner, of this city, and almost simultaneously by Professor Lippi, of Rome. Rosenmüller's work consisted mainly of an atlas of plates, nearly of the size of life, with a very meagre text. It was issued in three parts, under the title of "Chirurgisch-Anatomische Abbildungen für Aerzte und Wundaerzte." Tiedemann's great and unrivaled work on the arteries appeared at Heidelberg in 1821, and although it was not strictly surgical, it nevertheless belongs rather to this part of anatomy than to the purely descriptive. It was issued in folio with a quarto volume of text, and bears the title of "Tabulæ Arteriarum Corporis Humani." The illustrations are perfect representations of nature, and will never be likely to be surpassed by the efforts of succeeding anatomists.

In 1825 commenced a new era in surgical anatomy, signalized by the publication of the excellent monograph, in two octavo volumes, of M. Velpeau, then just entering upon his brilliant and successful career as a teacher and an author. The work, which was accompanied by an atlas of illustrations, and which bears the title of "Traité d'Anatomie Chirurgicale, ou d'Anatomie des Régions, considérée dans ses rapports avec la Chirurgie," passed through a second edition in 1833, and was translated and published at New York by Dr. Sterling, of that city. The American edition has long been out of print. Within a few years after the appearance of Velpeau's treatise, a work on surgical anatomy was issued by M. Milne Edwards, of Paris, since so well known as a physiologist and zoologist; and almost simultaneously with the French edition the profession was favored with a translation of the work by Mr. Coulson, of London. This translation was shortly after republished in this city, with notes by the late Professor Webster. The "Traité d'Anatomie Topographique," by Blandin, appeared at Paris in 1827, and was afterwards rendered into English by the late Dr. Sidney A. Doane, of New York. The work has no pretension to greatness, and has never passed to a second edition. Its plates, however, were admirable. In 1843 Nuhn, of Mannheim, published his "Handbuch der Chirurgischen Anatomie," a small production, unknown on this side of the Atlantic. In the same year J. E. Petréquin, of Lyons, favored his countrymen with an octavo volume on the subject, entitled "Traité d'Anatomie Medico-Chirurgicale et Topographique." Ross, of Leipsic, and Roser, of Tübingen, published their treatise on surgical anatomy, respectively, in 1848 and 1854.

Among the more recent publications on surgical anatomy, besides the treatise of Malgaigne, whose title heads this article, are the "Handbuch der Chirurgischen Anatomie," by F. Führer, of Hamburg, in two octavo volumes, with an atlas of twenty-two copperplates, (1857;) "Anatomie Chirurgicale Homalographique, ou Description et Figures des Principales Régions du Corps Humain," by E. Q. Le Gendre, (1858;) and the "Handbuch der Topographischen Anatomie," by Professor Hyrtl, of Vienna, in two large octavo volumes, the third and last edition of which appeared in 1857.

All mention has hitherto been purposely omitted of the works of British authors upon this subject, because it is our object to give a separate though brief notice of them here, apart from the productions of other nations. Few and imperfect as these works, with a single exception, are, it cannot be denied that they have exerted a most powerful influence upon the surgery and surgeons of Great Britain and the United States. The treatise of Allan Burns, entitled "Observations on the Surgical Anatomy of the Head and Neck," is known to every cultivator of anatomico-chirurgical science, and has long since become classical. Issued at Glasgow, in 1811, where Mr. Burns, then a young man, was a private lecturer on anatomy and surgery, it attracted at the time of its appearance universal attention among British teachers and practitioners, not less by the novelty and interest of the subject than by the charming style and deep eloquence infused into it by its able author. From that period dates a new era in surgical anatomy in the British empire, and also in the United States, where Mr. Burns's work was reprinted in 1823, under the supervision of that excellent anatomist, the late Granville Sharp Pattison, then Professor of Anatomy in the University of Maryland, and subsequently in the London University, the Jefferson Medical College, and, finally, in the University of New York. No one can read the work of Mr. Burns without being satisfied that he was a man of genius, and without being inspired with increased enthusiasm for the study of surgical anatomy and operative surgery. It was from an attentive study of its pages, everywhere so opulent in facts and suggestions, that our illustrious countryman, Dr. Mott, was seized with the conviction of the feasibility of ligating the innominate artery; a feat which he so admirably executed in 1818. The work of Mr. Burns deserves to be reproduced among us in a new form for the benefit of the rising generation of surgeons, many of whom, there is reason to believe, have never heard even of the name of the distinguished Scotchman, much less read his book. Had Mr. Burns not been cut off early in life by the ruthless hand of disease, the "Observations on the Surgical Anatomy of the Head and Neck" would no doubt have been followed by other volumes, until he would have traversed the entire body. As it is, we must be grateful for what he has left us.

We must not forget here to bestow a passing tribute of respect upon a laborious and accomplished Irish surgeon, who issued, cotemporaneously with Mr. Burns, a small treatise on Surgical Anatomy, the first part only of which, however, was ever published. We allude to the late Mr. Abraham Colles, of Dublin, for nearly fifty years an eminent teacher and practitioner in that city, and a gentleman who has enriched his profession by various valuable contributions. The work was republished in Philadelphia in 1820, and was for a long time a familiar companion with students in the dissecting-room.

Mention might also be made in this place of another admirable monograph by another Irish surgeon, namely, "The Surgical Anatomy of the Arteries," by the late Dr. Harrison, Professor of Anatomy in Dublin; but we are admonished to bring this article to a close, and shall therefore only add that, with the exception of Mr. Quain's splendid work on the same subject, a production of much later date, it is still the best treatise on the topographical relations of the arteries, in the English language.

Finally, in 1850 appeared the excellent work of Mr. Thomas Morton, entitled "The Surgical Anatomy of the Principal Regions of the Human Body," in one royal octavo volume of about four hundred closely printed pages, and illustrated by a number of superior plates and wood-cuts; the whole constituting, in our opinion, the most valuable and important contribution to topographical anatomy yet made by any British surgeon. The work is written in the clearest possible style, and reflects very great credit upon the knowledge and judgment of its late distinguished and lamented author. As a practical treatise, and a working-companion, it is far superior to any production of the kind with which we are acquainted.

The splendid volume on "Surgical Anatomy," by Mr. Joseph Maclise, was published about the same time as that of Mr. Morton, and was speedily reproduced in this city by the Messrs. Blanchard & Lea. It is accompanied by sixty-eight plates, drawn upon stone, and executed in admirable artistic style, the author having been his own lithographer. The treatise, as a whole, possesses many excellencies, but in clearness of description and practical adaptation it is inferior to that of Morton, and far less readable and instructive. It forms, nevertheless, an extremely valuable contribution to the science of topographical anatomy as taught in the schools of the present day.

Many excellent articles on surgical anatomy are contained in Todd's Cyclopedia of Anatomy and Physiology, written, for the most part, by men of distinguished ability and sound practical sense, and consequently worthy, as is everything else in that great work, of an attentive perusal.

The physicians of this country have not been unmindful of the value and importance of a knowledge of surgical anatomy, but they have always labored under the disadvantage of not having a good native work upon the subject; one which shall not, on the one hand, be too meagre, nor, on the other, too full for the purposes of the student; in other words, one that shall serve as a ready companion for the dissecting-room. That such a work, if properly constructed, would be well received by the American profession at this time, does not admit of any reasonable doubt, and it is equally certain that there are many men in the country well qualified to undertake it.

In concluding this brief, and, we fear, imperfect sketch of the history of surgical anatomy, into which we have been betrayed by the work of Malgaigne, we must not neglect to state that Dr. William Anderson, who, we believe, was a Scotchman, published, in 1822, at New York, the first part of a System of Surgical Anatomy; that Dr. N. R. Smith, of Baltimore, wrote the Surgical Anatomy of the Arteries, a quarto volume, of which a second edition was issued in 1835; and that Dr. William Darrach, of this city, is the author of a small brochure on the Surgical Anatomy of the Groin.

